# Review: An Update on CGRP Monoclonal Antibodies for the Preventive Treatment of Episodic Migraine

**DOI:** 10.1007/s11916-025-01365-4

**Published:** 2025-02-25

**Authors:** Kelly S Nicol, John G Burkett

**Affiliations:** Department of Neurology, Virginia Commonwealth University School of Medicine, 1101 E Marshall St Richmond, Richmond, VA USA

**Keywords:** Calcitonin gene-related peptide receptor antagonists, Episodic migraine, Erenumab, Fremanezumab, Galcanezumab, Eptinezumab

## Abstract

**Purpose of Review:**

CGRP targeting therapies have revolutionized the migraine preventive space, introducing novel migraine-specific therapies to improve headache care. Four monoclonal antibodies (mAbs) are approved for use in prevention of episodic migraines. Erenumab (AMG334), fremanezumab (TEV48125), and galcanezumab (LY2951742) are monthly subcutaneous injections, while eptinezumab (ALD403) provides an intravenous infusion option. This review aims to examine the clinical evidence for the safety and efficacy of CGRP-targeted mAbs in the prevention of episodic migraines with a focus on recent studies (2023–2024).

**Recent Findings:**

Long-term studies reveal ongoing safety and efficacy in recent literature for all 4 monoclonal antibodies. These investigations have built evidence for earlier access to CGRP treatment as they increase quality of life and reduce monthly migraine days while being better tolerated than non-specific migraine preventative therapies.

**Summary:**

These studies support the recent 2024 AHS consensus statement recommending CGRP monoclonal antibodies be considered as first-line preventive treatment in episodic migraine.

## Introduction

According to Burch et al. [1], the prevalence and impact of migraine in the United States affects almost 20% of the population and is female predominant. Attacks can cause severely disabling head pain generally characterized by light/sound sensitivity and/or nausea and can be accompanied by visual, sensorimotor, vestibular, and language symptoms. The headache characteristically lasts at least 4 hours and up to 3 days without treatment, with longer duration headaches being characterized as status migrainosus. Disability has historically been defined by scoring questionnaires, for example, the Migraine Disability Assessment Questionnaire (MIDAS) with a score of 11 or more as the threshold of significance [32] and the Headache Test (HIT-6) with a score of > 50% [2].

While migraine non-specific therapies offered relief in the late 1980s and early 1990s, a new drug target was identified when animal research identified neurogenic inflammation and abnormal trigeminovascular activation as key mechanisms in migraine. The CGRP peptide was identified as a potential culprit, recognized as the most ​​abundant neuropeptide in human trigeminal ganglia found in up to half of those neurons [3]. It was found to play a central role in downstream vasodilatory changes and peripheral and central sensitization [4, 5]. Two primary therapeutic approaches have since emerged: monoclonal antibodies (mAbs) and small-molecule antagonists (-gepants).

Literature up to 2022 has diligently summarized CGRP-mABs in this use case including the American Headache Society Society Consensus Statement 2021 [6] and Guidelines by the European Headache Federation reviewed 15 studies in episodic migraines [7]. There are also multiple review papers on this topic including one published in 2021 out of India looking at CGRP mAbs in episodic migraine [8] and another larger scale review from Cohen et.al in 2022 [9] which commented on mAbs and small molecules in both episodic migraines and chronic migraines. In 2024, CGRP-targeted migraine therapies are now a mainstay in modern-day migraine prevention and in this review, we will further specifically focus on the evidence for episodic migraine (< 15 days per month). Specifically, we intend to provide an update with tables showing episodic migraine studies with a focus on the primary endpoint of monthly migraine days (MMD) when possible, and then discuss a few specific trials, particularly more recent data that supports updates to recent guidelines (Table 1).

**Table 1:** Calcitonin Gene-Related Peptides (CGRP) monoclonal antibodies

Name	Approval Date	Pharmacokinetics (Route, Tmax, Half-life)	Dose and Frequency	Cost - Acquired from GoodRx®
Erenumab (AMG334)	May 2018	SC, 6 days, 28 days	70 mg, 140 mg, QM	$730 for 140mg/ml 1 sure click
Fremanezumab (TEV48125)	September 2018	SC,5 days, 32 days	225 mg QM, 675 mg QLT	$764 for 225mg/1.5 ml 1 autoinjector
Galcanezumab (LY2951742)	September 2018	SC,5 days, 27 days	120 mg, QM	$658 for 120mg/ml 1 pen
Eptinezumab (ALD403)	February 2020	IV, 1–3 h, 27 days	100 mg, 300 mg, QLT	$1827 (From Vyepti® website, cannot use GoodRx® Coupon)

Key Terms:

Pharmacokinetics: Includes Tmax (Time to peak drug concentration) and T1/2 (Half-life)

*SC* Subcutaneous, meaning the drug is injected under the skin

*IV* Intravenous, meaning the drug is injected directly into a vein

*QM* Monthly

*QLT* Quarterly

## Methods

Human and Animal Rights: All reported studies/experiments with human or animal subjects performed by the authors have been previously published and complied with all applicable ethical standards (including the Helsinki declaration and its amendments, institutional/national research committee standards, and international/national/institutional guidelines).

A comprehensive literature search was conducted using the PubMed database to identify relevant studies investigating the role of calcitonin gene-related peptide (CGRP) and CGRP receptor antagonists in the treatment of episodic migraine with monoclonal antibodies. The search was executed on March 3rd, 2024 and covered the period from January 1, 2010, to February 20, 2024. The search terms and Medical Subject Headings (MeSH) used were as follows: a PubMed search with the following criteria "Calcitonin Gene-Related Peptide"[MAJR] OR "Calcitonin Gene-Related Peptide Receptor Antagonists"[MAJR] OR "Receptors, Calcitonin Gene-Related Peptide"[MAJR] OR "calcitonin gene-related peptide"[ti] OR "calcitonin gene-related peptides"[ti] OR CGRP[ti] OR CGRP[ot]) AND (("Migraine Disorders"[Mesh] OR migraine[ti] OR migraines[ti]) AND episodic) AND (monoclonal[ti] OR "Antibodies, Monoclonal"[MeSH] OR "Antibodies, Monoclonal"[nm]) AND (2010:2024[pdat]). Inclusion criteria included research articles, clinical trials, randomized control trials, reviews, and meta-analyses focusing on CGRP or CGRP receptor antagonists in the context of episodic migraine, this was narrowed to randomized control trials and clinical trials to identify primary sources and open-label extension studies.

## Discussion

### Erenumab

In a comparison of preventive treatments within a German population, erenumab was directly compared to topiramate in a randomized, double-blind, double-dummy phase 4 trial which was designed to strategically account for nocebo/placebo effect attributable to therapy. This study, HER-MES, had 777 participants and observed for erenumab effect compared with the current standard of care topiramate therapy. Agent selection was important as topiramate is one of the most commonly chosen first-line preventative drugs. Erenumab gave 55% of patients ≥ 50 reductions in monthly migraine days versus only 31% in the topiramate group [19]. The authors suggest this may support earlier access to erenumab (Table 2).

**Table 2:** Summary of erenumab clinical trials for episodic migraine and open-label studies

References	Title	Phase	Study Period/ n	Primary End Points
Sun et al. 2016 [10]	Safety and efficacy of AMG 334 for prevention of episodic migraine: a randomized, double-blind, placebo-controlled phase 2 trial	II	3 months/ n = 483	MMD reduction: Placebo: −2.3 70mg: −3.4 (p = 0.021) 7mg and 21mg not significant
Goadsby et al. 2017 [11]	A Controlled Trial of Erenumab for Episodic Migraine	III	4–6 months/ n = 955	MMD reduction: Placebo: − 1.8 70 mg: − 3.2 (*p* < 0.001) 140 mg: − 3.7 (*p* < 0.001)
Dodick et al. 2018 [12]	ARISE: A Phase 3 randomized trial of Erenumab for episodic migraine	III	3 months/ n = 577	MMD reduction: Placebo: − 1.8 70 mg: − 2.9 (p < 0.001)
Reuter et al. 2018 [13]	LIBERTY: Efficacy and tolerability of Erenumab in patients with episodic migraine in whom two-to-four previous preventative treatments were unsuccessful: a randomized double-blind, placebo-controlled phase 3b study	IIIb	3 months/ n = 246	>/=50% reduction rate of MMD: Placebo: 14% 140 mg: 30% (p = 0.002)
Sakai et al. 2019 [14]	A Randomized phase 2 Study of Erenumab for the Prevention of episodic migraine in Japanese adults	II	4–6 months/ n = 475	MMD reduction: Placebo: 0.06 28 mg: − 1.25 (p = 0.004) 70 mg: − 2.31 (p < 0.001) 140 mg: − 1.89 (p < 0.001)
Goadsby et al. 2020 [15]	One-year sustained efficacy of Erenumab in episodic migraine: results of STRIVE study	III	12 months/ *n* = 845	MMD reduction: 70 mg: − 4.2 140 mg: − 4.6
Goadsby et al. 2021 [16]	Long-term Efficacy and Safety of Ereneumab: Results from 64 weeks of the LIBERTY study	III	64 weeks/ n = 204	50% or greater reduction rate of MMD: 44.3%
Takeshima et al. 2021 [17]	Erenumab treatment for migraine prevention in Japanese patients: Efficacy and Safety results from a phase 3 randomized, double-blind, placebo-controlled Study	III	4–6 months/ n = 261	MMD reduction: Placebo: − 1.98 70 mg − 3.60 (p < 0.001)
Wang et al. 2021 [18]	Randomized, controlled trial of Erenumab for the prevention of episodic migraine in patients from Asia, the Middle East, and Latin America: The EMPOwER study	III	3 months/ n = 900	MMD reduction: Placebo: − 3.1 70 mg: − 4.2 (p = 0.002) 140 mg: − 4.8 (p < 0.001)
Reuter et al. 2022 [19]	Erenumab versus topiramate for the prevention of migraine - a randomised, double-blind, active-controlled phase 4 trial HER-MES	IV	24 weeks/ n = 777	Rate of discontinuation: Erenumab 70mg/140mg 10.6% Topiramate: 38.9% (p < .001) 50% or greater reduction rate of MMD: Erenumab: 55% Topiramate: 31.2% (p < .001)
Pozo-Rosich et al. 2024 [20]	Early Use of Erenumab vs Nonspecific Oral Migraine Preventives: The APPRAISE Randomized Clinical Trial	OLEP	**12 months / n = 621	50% or greater reduction rate of MMD Ereneumab: 56.2% OMPM: 16.8% P < .001
Reuter et al. 2024 [21]	Efficacy and Safety of Erenumab in Participants with Episodic migraine in whom 2–4 prior preventative treatments had failed: Liberty 3-year study	OLEP	**3 years/ n = 168	MMD reduction at weeks 61–64: Continuous Erenumab 140mg: −3.8 Post week 12 placebo switched to erenumab: −3.6

Key Terms:

** Time beyond primary study - open-label duration only

OMPM = oral preventative medication

The APPRAISE trial is an open-label study published May 2024. It showed a sustained one year benefit of using erenumab compared to non-specific oral migraine preventative medications in 621 patients. This was the first multicenter randomized control trial to practically compare an anti-CGRP mAb vs. traditional oral therapies in patients who had failed 1–2 preventative agents. The results established evidence for the earlier use of CGRP therapies to avoid chronification of migraine. APPRAISE showed 56% of patients treated with erenumab reduced monthly migraine days by over half, while in non-specific medications, only 16% achieved this reduction. In addition, patients’ subjective reports that erenumab was more efficacious was aligned with high PGIC (patient global impression of change) scales ≥ 5 at one year tallying 76% in the CGRP group vs. lower scores of 18% in the non-specific treatment group. Beyond establishing efficacy, this study showed ongoing adherence to erenumab after 1 year with only 2.2% requiring a medication switch vs.the non-specific group at 34.6%. Discontinuation rates with erenumab were also favorable with 2.9% electing to stop treatment due to adverse events versus 23.3% from the non-specific medications group. Ultimately, no new safety risks were identified in the erenumab group compared to prior literature. Erenumab showed sustained long-term benefit starting early at 4 weeks and continuing through the 12 month time period. This data demonstrates additional findings that support for erenumab to be offered earlier in a patient’s treatment course [20].

The LIBERTY Study observed efficacy and safety for the use of erenumab in episodic migraine, in 2024, an update to this was published after in which 168 participants could enter the open label extension phase (OLEP) to receive 140mg erenumab once monthly for 3 years with ≥ 50% reduction in monthly migraine days, change from baseline, and tolerability/safety as the primary metrics. For the continuous erenumab group, 35/117 were ≥ 50% responders after 12 weeks of double blind treatment around 3/4th of that group had continued response of ≥ 50% reduction in monthly migraine days at least half of OLEP, which put the overall sustained benefit number at 26/117. However, for those who had enough time to benefit from erenumab in the initial 12 week treatment, when kept on erenumab, 20.7% converted to having ≥ 50% reduction in monthly migraine days showing if given more time on the medication, this improved to 43/117 response rate. For those who had been placebo treated then switched to ereneumab, but not responded by the 12 weeks time point, ongoing therapy converted 40.5% of participants into responders in the OLEP visits. Therefore, from the start of the study to the end of the total participants the mean (SD) MMD change from baseline over 3 years was − 4.4 [3.9] days at week 168. The safety events and adverse drug effect profile was similar to earlier studies [21].

At present, there are no migraine-specific preventive therapies approved for children or adolescents with migraine, but erenumab is under active investigation for use in the pediatric population ages 9–17. Hershey et al., conducted a phase 1 randomized open-label multiple-dose study in children and adolescents published in February 2024. They did an analysis of treatment-emergent events, vital signs, ECG, clinical labs, neurologic assessments, and pharmacokinetic concentrations of the drug. Although this was a small study with 53 participants, the pharmacokinetics and safety of erenumab in pediatric patients was consistent with adults allowing for weight differences. While efficacy was not evaluated in this study, an exploratory endpoint of PedMIDAS total score (modified pediatric migraine disability assessment) did show erenumab reduced migraine disability [22], suggesting erenumab may be safe and effective for use in the pediatric population.

### Galcanezumab

Galcanezumab has been shown in many studies to be both safe and effective since 2022, this has been demonstrated in multiple international populations highlighting its potential impact on migraine disability world-wide. In 2022, Kim et al, published a population-based study in South Korean patients as a post-hoc analysis of the EVOLVE-2 phase 3 trial of galcanezumab. Specifically, 98 participants in the intent-to-treat population were compared to placebo with low and high-dose galcanezumab. In a 6 month period, only the higher 240mg dose of galcanezumab resulted in a significant reduction from baseline in mean number of monthly migraine days, however, in the all-patients population, both doses were effective. Acute medication use was also evaluated and showed both doses resulted in a significantly higher reduction from baseline with acute medication use compared to placebo, which was consistent with the all-patient’s population. Finally, for patient-reported outcomes, galcanezumab low dose treatment resulted in an increase from baseline mean MSQ RF-R score compared to placebo. Interestingly, the higher dose was not significantly different from placebo; however, in the all-patient group both doses were effective. In terms of MIDAs and PGI-Scores scores in the Korean population, neither dose made a significant difference which again was contrary to the all-patients group in which both doses significantly reduced scores [29] (Table 3).

**Table 3:** Summary of galcanezumab clinical trials for episodic migraine and open-label studies

References	Title	Phase	Study period/ n	Primary Endpoints
Dodick et al. 2014 [23]	Safety and efficacy of LY2951742 a monoclonal antibody to calcitonin gene-related peptide, for prevention of migraine: A phase 2, randomized, double-blind, placebo-controlled study.	II	3 months / n = 218	MMD reduction: Placebo: −3.0 150mg: −4.2 (p = .003)
Stauffer et al. 2018 [24]	Evaluation of galcanezumab for the prevention of episodic migraine: the EVOLVE-1 randomized clinical trial	III	6 months / n = 858	MMD reduction: Placebo:−2.8 120mg: −4.7 240mg: −3.36 (both p = < .001)
Skljarevski et al. 2018 [25]	Efficacy and safety of galcanezumab for prevention of episodic migraine: results of the EVOLVE-2 phase 3 randomized controlled clinical trial	III	6 months / n = 915	MMD reduction: Placebo: −2.3 120mg: −4.3 240mg: −4.2 (both p = < .001)
Sakai et al. 2020 [26]	Efficacy and safety of galcanezumab for prevention of migraine headache in Japanese patients with episodic migraine: A phase 2 randomized controlled clinical trial.	II	6 months / n = 915	MMD reduction: Placebo: −0.59 120mg: −3.6 240mg: −3.36 (both p = < .001)
Mulleners et al. 2020 [27]	Safety and efficacy of galcanezumab in patients for whom previous migraine preventative medication from 2–4 categories had failed (CONQUER): A multicenter, randomized, double-blind, placebo-controlled, phase 3b trial	III	3 months / n = 462	MMD reduction: Placebo: −1.0 120mg: −4.1 (p = < .0001)
Reuter et al. 2021 [28]	Galcanezumab in patients with multiple previous migraine preventative medication category failures: results from the open-label period of the CONQUER trial	III + OLEP	3 months 3 months ** / n = 449	MMD reduction: Placebo: −4.5 Treatment: −3.8
Kim et al. 2023 [29]	Efficacy and Safety of Galcanezumab as a Preventive Treatment for Episodic Migraine in South Korean Patients: A Post-Hoc Analysis of a Phase 3 Clinical Trial	III	6 months/ n = 98	50% or greater reduction rate of MMD: Placebo: 32.9 120mg: 54.4, odds ratio 2.43, p = .030 240mg: 56.1, odds ratio 2.6, p = .019
Zhou et al. 2023 [30]	Galcanezumab in patients with episodic migraine: results from the open-label period of the phase 3 PERSIST study	III + OLEP	3 months 3 month** /n = 484	MMD reduction after 3 months OLEP Placebo switch to galcanezumab:: −4.56 Continuous galcanezumab: −4.62
Shibata et al. 2023 [31]	Galcanezumab Efficacy Through the Dosing Interval in Japanese Patients with Episodic Migraine: Post Hoc Analysis of a Phase 2 Randomized Trial	II + posthoc	6 months/ n = 459	≤ 9% had an increase of ≥ 2 migraine headache days per week from week 2 to week 4 in ≥ 2 treatment months in 240mg, 120mg and placebo treatment groups
Lipton et al. 2023 [32]	Changes in migraine interictal burden following treatment with galcanezumab: Results from a phase III randomized, placebo-controlled study	III + OLEP	3 month 3 month ** / n = 462	Percentage of all patients with severe interictal burden decreased from 59% (137/232) at baseline to 27% (58/217) at Month 6 (episodic migraine from 51% [70/137] to 23% [30/131])

Key Terms:

** Time beyond primary study - open-label duration only

In 2023 Zhou et al, published on the results of galcanezumab’s use in the open-label phase of the phase 3 PERSIST study which looked at long-term efficacy and safety of galcanezumab in patients from 26 centers in China, 20 in India, and 4 within Russia. There were multiple study periods including a screening/washout followed by a baseline period. Then a 3-month, randomized, double-blind, placebo-controlled phase, followed by a 3 month open-label portion with one group receiving continuous galcanezumab, another with placebo initially, then started on galcanezumab for the 3-month open-label extension period, then followed by a 4 month post treatment phase. Overall, the continuous galcanezumab group showed ongoing efficacy, with average MMDs significantly decreasing from baseline and more specifically, those who had ≥ 50% reduction at month 3 had sustained response even at month 6. Moreover, in the placebo-to-galcanezumab group, participants saw immediate impact in reduced MMDs that was maintained at month 6 with a mean reduction of 4.56 days [30].

The sustained efficacy in monthly dosing interval of galcanezumab in a Japanese population (n = 459) was investigated as a post-hoc analysis of a phase 2, randomized, placebo-controlled trial which took place over 3 years. Shibata et al. showed that over a 6 month time course, galcanezumab had ongoing efficacy in significantly reducing monthly migraine headache days and there was not evidence of wearing-off effect month-to-month, with ≤ 9% having an increase of at least two migraine headache days per week from week 2 to week 4 in at least two of the treatment months, which was consistent across 120mg, 240mg and placebo dosing regimens [31].

Finally, Lipton et al., 2023 recently evaluated the burden of disability in the interictal period in the CONQUER trial. This was a double-blind study, 3 months duration, with 462 patients total. After galcanezumab was used for 3 months of open-label data collection, mean interictal burden was assessed with the Migraine Interictal Burden Scale (MIBS-4). The study showed treatment with galcanezumab significantly reduced MIBS-4 score versus placebo at month 3 with additional improvement at month 6, suggesting sustained improvement in quality of life [32].

### Fremanezumab

There have been several notable open-label studies for fremanezumab that support long-term safety and tolerability data. In 2021, Sakai et al investigated fremanezumab treatment in a 52-week multi-center, randomized, parallel-group study in Japanese patients. While Japanese patients were included in HALO-EM and HALO extension trials with demonstrated efficacy/safety, this study aimed to further assess those metrics in 16 patients total, 8 participants in each group receiving either monthly or quarterly drug administration. The most common reactions were nasopharyngitis and infection-site reactions consistent with prior data. In addition, anti-drug antibody development was rare and there were no drug-related serious adverse events or deaths. For efficacy, the data was not separated for EM and CM but did show ongoing significant reductions in average monthly migraine days, headache days of at least moderate severity, headache days of any severity, and use of any acute headache medication regardless of administration pattern. This cohort had reduction in baseline disability with reduced MIDAS score at month 12 with mean scores going from 20.5+/- 22.1 down to 11.4 +/−25.4 in the monthly administration group and for the quarterly, mean scores dropped from 12.6 +/- 13.7 down to 11.9 +/- 21.6 [37] (Table 4).

**Table 4:** Summary of fremanezumab clinical trials for episodic migraine and open-label studies

References	Title	Phase	Study Period/ n	Primary Endpoints
Bigal et al. 2015 [33]	Safety, tolerability, and efficacy of TEV-48125 for preventative treatment of episodic migraine: a multicentre, randomized, double-blind, placebo-controlled phase 2b study.	II	3 months / n = 297	Least square mean decrease in MMD: Placebo: −3.46 225 mg: −6.27 675 mg: −6.09 (both p < 0.0001)
Ferrari et al. 2019 [34]	Fremanezumab versus placebo for migraine prevention in patients with documented failure to up to four migraine preventative medication classes (FOCUS): a randomized, double-blind, placebo-controlled phase 3b trial.	III	3 months / n = 838	MMD reduction: Placebo: 0.6 225 mg: −4.1 675 mg: −3.7 (p < 0.001)
Dodick et al. 2018 [35]	Effect of fremanezumab compared with placebo for prevention of episodic migraine: a randomized clinical trial (HALO-EM)	III	6 months / n = 777	MMD reduction: Placebo: −2.2 225mg: −3.7 675mg: −3.4 (both p < 0.001)
Goadsby et al. 2020 [36]	Long-term safety, tolerability, and efficacy of fremanezumab in migraine: a randomized study (HALO-EM)	III	12 months / n = 780	MMD reduction: 225mg: −5.1 675mg: −5.2
Sakai et al. 2021 [37]	Long-Term Safety and Tolerability of Fremanezumab for Migraine Preventive Treatment in Japanese Outpatients: A Multicenter, Randomized, Open-Label Study	OLEP	52 week / n = 23/22	MMD reduction at week 52: Monthly: −5.9 Quarterly: −1.6
Ashina et al. 2021 [38]	Efficacy and safety of fremanezumab in patients with episodic and chronic migraine with documented inadequate response to 2 to 4 classes of migraine preventive medications over 6 months of treatment in the phase 3b FOCUS study	IIIb	12 week 12 week** n = 772	MMD reduction: Initial placebo then 225mg monthly (3 rounds): −4.7 Quarterly: −5.1 Monthly: −5.5
Ashina et al. 2023 [40]	Real-world effectiveness of fremanezumab for the preventive treatment of migraine: Interim analysis of the pan-European, prospective, observational, phase 4 PEARL study	IV	6 month / n = 72	50% or greater reduction rate of MMD: Episodic Migraineurs: 69.4%

Key Terms:

** Time beyond primary study - open-label duration only

The FOCUS phase 3b study resulted in several additional publications as open-label extension studies.

In 2021, Ashina et al., looked at a 12 week open-label extension after the initial 12 week trial where placebo patients were then given 225mg monthly for 3 months and compared to those continuously on quarterly or monthly dosing. They found ≥ 50% reduction in monthly migraine days in 38% of those initially treated with placebo, continuous quarterly drug administration had 45% hit this benchmark, and 20% with continuous monthly administration did as well. When the improvement criteria became more stringent looking at ≥ 75% reduction in monthly migraine days, 16% in the initial placebo group reached this benchmark, while 15% of the quarterly fremanezumab group and 20% of the monthly drug administration group did as well. These results suggest sustained efficacy up to 6 months and as well as showing overall low rates of adverse events < 1% in all groups [38] Spierings et al., 2021 also conducted an open-label extension study to assess patient-reported outcomes in 313 episodic migraine patients. Patients had completed 12-weeks of double-blind treatment then entered an additional 12 weeks of OLE and received three monthly doses of fremanezumab at 225mg. At 6 months, although the team did not separate chronic migraine from episodic migraine data, they found overall significant improvement in RFR, RFP, and EF portions of the MSQoL questionnaire, the EeQ-5D-5L questionnaire, the WPAI questionnaire, and the PHQ-9. Overall, fremanezumab had a favorable improvement in quality of life, depression, and productivity [39].

The ongoing Pan-European Real Life (PEARL) study published an update in 2023 of its phase 4 data evaluating effectiveness and safety of fremanezumab in 72 episodic migraine patients. It showed 69% had ≥ 50% reduction in monthly migraine days within 6 months of starting fremanezumab. Expanding out to 12 months with n = 13 so far, participants had a decrease of 8.3 MMD (monthly migraine days) total as well as continued reduction in acute migraine medication use, migraine-related disability according to MIDAS/HIT-6 scores, and peak headache severity on an 11-point numerical rating scale. This study will ultimately conclude at 24 months and is being conducted in 87 sites across 11 European countries [40].

### Eptinezumab

In 2023, Goadsby et al. published a phase 3b, randomized, double-blind placebo-controlled DELIVER clinical trial which investigated the role of eptinezumab in reducing not only migraine frequency but also overall patient health and quality of life. Data was collected from USA and Eupropean sites to include 17 countries and 96 study locations. The team looked at data up to 24 weeks of use, and they found that by week 12, EQ-5D-5L scores measuring overall patient health, HIT-6 scores, MIDASs scores, and PGIC scores all improved [44]. These results provide evidence that CGRP therapy for episodic migraine is promoting multidimensional well-being and improved quality of life beyond decreasing monthly migraine days (Table 5).

**Table 5:** Summary of eptinezumab clinical trials for episodic migraine

References	Title	Phase	Study Period/ n	Primary Endpoints
Dodick et al. 2014 [41]	Safety and Efficacy of ALD403 an antibody to calcitonin gene-related peptide for the prevention of frequent episodic migraine: randomized, double-blind placebo-controlled exploratory phase 2 trial	II	3 months / n = 174	MMD reduction: Placebo: −4.6 1000mg: −5.6 (p = .03) >= 75% migraine responder rate: placebo 20.7%
Ashina et al. 2020 [42]	Eptinezumab in episodic migraine: a randomized, double-blind, placebo-controlled study (PROMISE-1)	III	3 months / n = 888	MMD reduction: Placebo: −3.2 30mg: −4.0 (p = .0046) 100mg: −3.9 (p = .0182) 300mg: −4.3 (p = .0001)
Smith et al. 2020 [43]	Eptinezumab for the prevention of episodic migraine: sustained effect through 1 year of treatment in the PROMISE-1 study	III	48 weeks / n = 888	MMD reduction: Placebo: −4.1 30mg: −5.0 (95% CI −1.61 to − .11), 100mg: −4.5 (95% CI −1.31 to 0.37), 300mg: −5.3 (95% CI −1.95 to − .46)
Poszo-Rosich et al. 2024 [46]	Eptinezumab Demonstrated Efficacy Regardless of Prior Preventive Migraine Treatment Failure Type: Post Hoc Analyses of the DELIVER Study	IIIb	24 weeks/ n = 890	24 week MMD reduction Placebo: −2.0–2.6 100mg or 300mg: −5.0-6.2

Pharmacokinetic profiles at population-specific level are an active area of investigation. Current data suggests that monoclonal antibodies have similar drug concentrations/concentration-time curves, volume of distribution, and clearance across demographics and variable characteristics in subgroup analysis, but more specific population evaluation is ongoing. Xue-Ning Li et al. evaluated 20 healthy Chinese individuals compared to non-asian populations over 12 weeks while receiving eptinezumab. Over both groups it was well tolerated with no novel safety events found. Mean plasma concentrations were similar regardless of dosing (100mg vs. 300mg) and the half life remained in an average range of 22.5–28.1 days. Notably, as with any antibody therapy, there is potential for development of anti-drug antibodies - and one patient in the 100mg dose cohort did develop anti-drug antibodies, but no neutralizing antibodies were seen. Anti-drug and neutralizing antibodies remain a topic of ongoing study for CGRP mABs[45].

Eptinezumab anti-CGRP therapy is also being evaluated in its therapeutic benefit for episodic migraine globally. In the DELIVER study, published January 2024, eptinezumab was compared to placebo in a multi-center parallel-group, double-blind, randomized phase 3b clinical study. It was done at 96 locations over 24 weeks in Europe and the US and had 890 patients (eptinezumab 100 mg, n = 299; eptinezumab 300 mg, n = 293; placebo, n = 298). Within this group, 633 (71.1%) patients had previously failed topiramate, 538 (60.4%) who previously failed beta blockers, 508 (57.1%) previously failing amitriptyline and 333 (37.4%) who had previously failed flunarizine. Overall, eptinezumab achieved a greater decrease in MMD starting at weeks 1–4 and extending out through the 24 week period. Half of the patients treated with 300mg eptinezumab achieved ≥ 50% response vs. <15% response in the placebo group. In the subgroup analysis, this effect was observed regardless of which of the 4 non-specific migraine medications patients had previously failed [46].

## Conclusions

### Future Directions

The next few years will provide further data on CGRP targeting antibodies as well, with a search of ClinicalTrials.gov showing ongoing research on erenumab focusing on microRNA profiles, identification of genetic and biomarker predictors to personalize treatment, and looking at efficacy in the pediatric populationthe. Additionally, there is active research of galcanezumab its effects on breast milk. For fremanezumab, future studies are investigating its effects on interictal migraine burden. Finally, eptinezumab is being examined in the context of refractory migraine cases and will look at the induction properties of PACAP-38.

### American Headache Society Consensus Statement Update

Anti-CGRP monoclonal antibodies are clearly an effective tool for the treatment of migraine based on the breadth of studies showing their utility. Since the initial 2021 American Headache Society (AHS) consensus statement, typically CGRP agents have been used in adult patients who failed to tolerate or respond to at least 2 migraine non-specific medications. Clinical trials, including phases II and III, have consistently shown that CGRP mAbs are both effective and well-tolerated [6]. As more research accumulates, the most pressing current question facing providers, insurers and patients are in regards to access and whether sooner access is warranted.

More recent studies that have evaluated the anti-CGRP monoclonal antibodies as a class have also yielded some notable findings supporting earlier access. A more recent 2024 systematic review and meta-analysis of eptinezumab, fremanezumab, galcanezumab, and erenumab, showed that when compared to placebo, there was statistically significant differences decreasing numbers of patients with medication overuse or medication overuse headache when using triptans or multiple drugs, albeit, not simple analgesics [47].

Roblee et. al looked at RCTs of CGRP mAbs, topiramate and divalproex in a meta-analysis, to evaluate the evidence for stepped care, which is the current setup for most insurance plans, requiring multiple preventive trials before CGRP mAbs may be used. The study found that there is high certainty that the mAbs are more effective than placebo, as are topiramate and divalproex, but that these oral anti-epileptic options are less tolerated and supporting first line preventive access to CGRP mAbs for patients, as they are likely just as effective, with better side effect profiles [48]. Finally, a 2024 prospective study with the largest to date study population of 5818 patients showed that over half of patients treated with CGRP mAbs had a 50% or more reduction in monthly headache days. Notably, it also showed that unilateral pain, less monthly migraine days and lower disability at baseline was more likely to predict a better treatment response and thus suggests that earlier access to this treatment option would increase chance of success, treating patients before migraines become more highly disabling and frequent [49].

As a result of the ongoing accumulation of data, the AHS updated their 2024 consensus statement to now recommend that monoclonal antibodies targeting CGRP or its receptor, including erenumab, fremanezumab, galcanezumab, or eptinezumab, should be considered as first-line options in patients with episodic migraine with or without aura (4–14 MMDs) based upon ICHD-3 criteria with at least moderate disability (MIDAS score ≥ 11 or HIT-6 score > 50) [50]. How insurance plans may adjust in the future is yet to be determined, but for patients suffering from migraine, easier access to effective, well-tolerated medications for migraine prevention would be welcomed.

## Data Availability

No datasets were generated or analysed during the current study.
